# Successful surgical treatment of brachial aneurysm associated with arteriovenous fistula for hemodialysis

**DOI:** 10.1093/jscr/rjad213

**Published:** 2023-04-21

**Authors:** Shusaku Maeda, Masahiro Ryugo, Kana Shibata, Yukinori Kashiyama, Hiroki Nakatsuji, Yasushi Tsutsumi, Osamu Monta

**Affiliations:** Department of Cardiovascular Surgery, Fukui Cardiovascular Center, Fukui, Fukui, Japan; Department of Cardiovascular Surgery, Fukui Cardiovascular Center, Fukui, Fukui, Japan; Department of Cardiovascular Surgery, Fukui Cardiovascular Center, Fukui, Fukui, Japan; Department of Cardiovascular Surgery, Fukui Cardiovascular Center, Fukui, Fukui, Japan; Department of Cardiovascular Surgery, Fukui Cardiovascular Center, Fukui, Fukui, Japan; Department of Cardiovascular Surgery, Fukui Cardiovascular Center, Fukui, Fukui, Japan; Department of Cardiovascular Surgery, Fukui Cardiovascular Center, Fukui, Fukui, Japan

## Abstract

A 58-year-old female who underwent renal transplantation and closure of arteriovenous fistula (AVF) for hemodialysis at age 24 was presented with left forearm pain and cyanosis. Computed tomography revealed an obstructed true brachial aneurysm at the anterior aspect of the elbow joint. Under a diagnosis of true brachial aneurysm associated with AVF, aneurysm resection and brachial to ulnar artery bypass grafting using a reversed great saphenous vein were performed. To prevent graft occlusion due to elbow flexion, it was routed through the ulnar side of the elbow joint. One year after surgery, the patient was asymptomatic with a patent graft.

## INTRODUCTION

A true brachial aneurysm is a very rare complication of an arteriovenous fistula (AVF) created for hemodialysis access [1]. Reported here are details of a patient with a true brachial aneurysm associated with an AVF for hemodialysis who underwent a successful surgery.

## CASE REPORT

A 58-year-old female was presented with pain and cyanosis of the left forearm, and referred to our hospital. At the age of 24 years, she suffered from end-stage renal failure due to IgA glomerulonephritis and underwent AVF creation for hemodialysis. Later that same year, she received living-donor renal transplantation and then the AVF was closed. At the age of 56, pain and cyanosis of the left forearm developed, and ultrasound findings led to a diagnosis of true brachial aneurysm. Antiplatelet therapy with aspirin was started because the symptoms were mild and intermittent. However, symptoms gradually worsened and she was referred to our hospital for surgical treatment.

**Figure 1:**
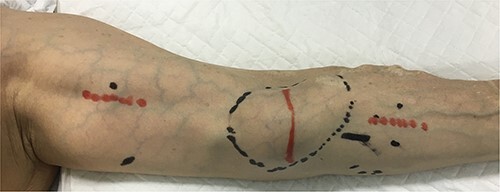
The initial physical examination showed a hard mass (black circle) at the left antecubital fossa of the volar aspect. Red lines indicate planned incision lines for surgery.

**Figure 2:**
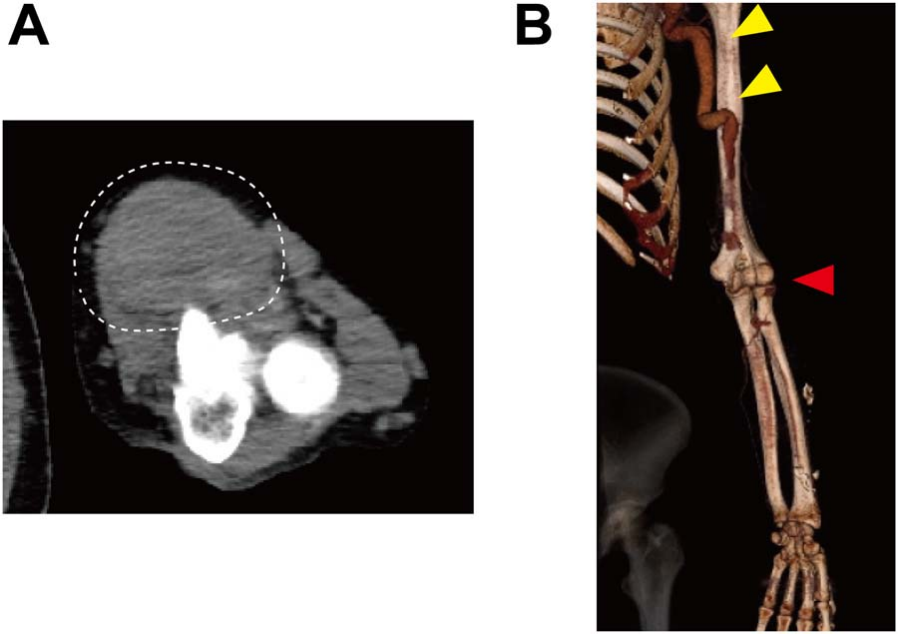
Preoperative contrast CT showing (**A**) a true brachial aneurysm at the anterior aspect of the elbow joint, which was obstructed by a thrombus (white dashed circle), and (**B**) the dilated brachial artery (yellow arrows). Red arrow indicates the level of the true brachial aneurysm.

Upon admission, the patient received immunosuppressive treatment with prednisolone (5 mg/day), tacrolimus (1.5 mg/day) and everolimus (0.5 mg/day) for renal transplantation. A physical examination revealed a hard mass in the left antecubital fossa of the volar aspect (Fig. 1), and contrast computed tomography (CT) showed a true brachial aneurysm measuring 32 × 42 mm in diameter at the anterior aspect of the elbow joint (Fig. 2A). In addition, the brachial artery in the proximal site of the aneurysm was dilated to 15 mm in diameter (Fig. 2B). Although the aneurysm and radial artery were completely obstructed with a thrombus, the ulnar artery was enhanced with blood supply from a collateral artery. These findings suggested that the pain and cyanosis symptoms were caused by ischemia or distal embolism. 

Under a diagnosis of symptomatic true brachial aneurysm, a procedure for resection of the aneurysm and bypass grafting to the ulnar artery was scheduled. Intraoperative findings revealed a brachial aneurysm just proximal to the brachial bifurcation. Following incision of the aneurysm, the orifices of the afferent and efferent arteries were closed, then the thrombus was removed for volume reduction. Brachial to ulnar artery bypass grafting using a reversed great saphenous vein was subsequently performed. To prevent graft occlusion due to elbow flexion, the graft was routed through the ulnar side of the elbow joint. Accounting for natural graft routing, a distal anastomosis was made in an end-to-side manner, whereas a proximal anastomosis was also done in an end-to-side manner because of a size mismatch between the dilated brachial artery and saphenous vein graft.

**Figure 3:**
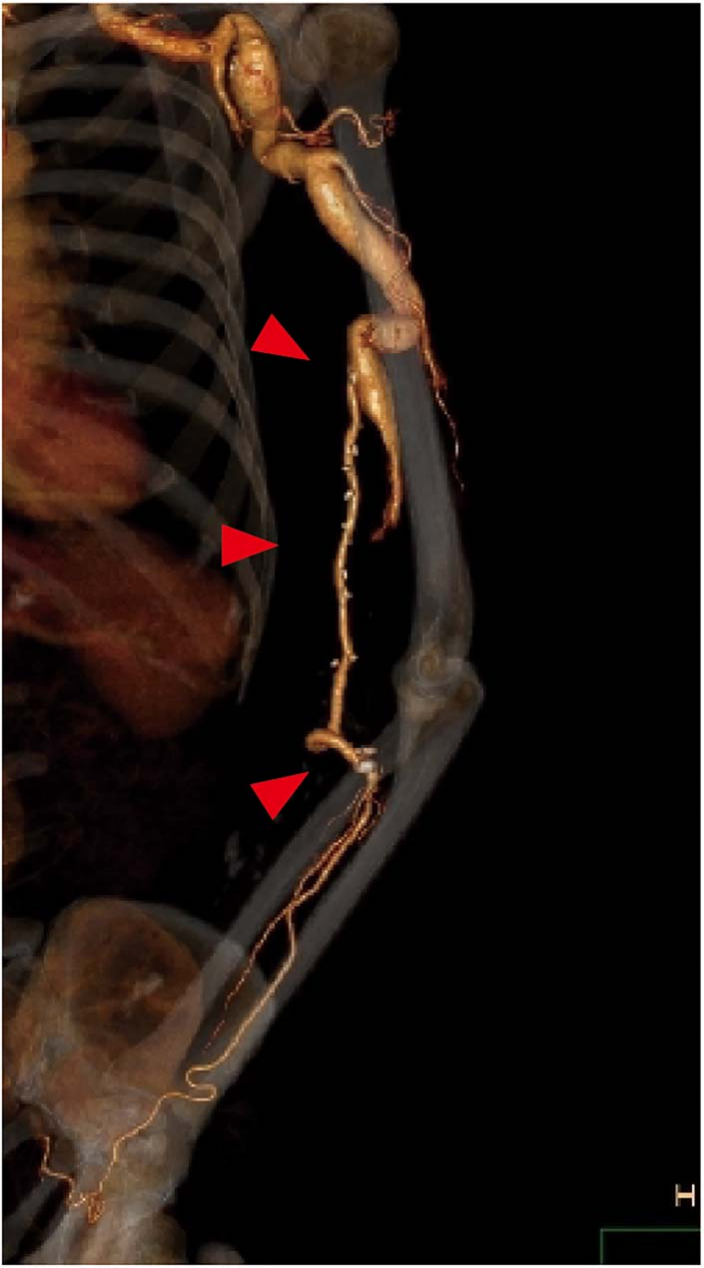
Postoperative contrast CT showing the patent great saphenous vein graft (red arrows) anastomosed from the brachial to ulnar artery. To prevent graft occlusion due to elbow flexion, the graft was routed through the ulnar side of the elbow.

Follow-up CT showed patency of the great saphenous vein graft (Fig. 3), and vascular Doppler ultrasound indicated a sufficient palpable pulse in the radial artery, with the latter finding confirmed when the elbow was flexed 90 degrees. The postoperative course was uneventful. At 1 year after surgery, the patient was asymptomatic and graft patency was confirmed by an ultrasound examination.

## DISCUSSION

The true brachial aneurysm associated with hemodialysis access was reported in a very limited number of patients [1]. A review of cases of a true brachial aneurysm associated with hemodialysis access noted that the mean period from creation of AVF to diagnosis of brachial aneurysm was 21 years and from renal transplantation to diagnosis was 14 years. These findings indicate that the development of a true brachial aneurysm occurs over a very long period. The number of patients receiving hemodialysis has been rising and prognosis following kidney transplantation improving [2], which has increased the probability of encountering a patient with a true brachial aneurysm associated with AVF in daily practice.

The mechanism underlying aneurysmal development in patients with AVF is not fully understood, though Basile *et al*. [3] reported the following in this regard. Creation of an AVF raises the rate of blood flow in the brachial artery, which increases arterial shear stress. To compensate for that increase, vascular endothelium releases vascular relaxing factors including nitric oxide, resulting in arterial dilatation. In addition, reactive oxygen species upregulated by increased shear stress may cause fragmentation of the internal elastic lamina through upregulation of matrix metalloproteinase. Further arterial dilatation can also occur even after closure of an AVF, because of damage to the elastic network of arterial walls. In addition, immunosuppressant drugs given after renal transplantation have been suggested to be associated with continued arterial dilatation [4]. The present patient developed symptoms at 30 years after renal transplantation and AVF closure, and was given three immunosuppressant drugs. Careful follow-up examinations of the brachial artery using ultrasound may be recommended for patients with a closed AVF long after undergoing renal transplantation.

Resection of the aneurysm and revascularization using a reversed great saphenous vein graft is the gold standard treatment for this disease [4]. Only exclusion of the aneurysm can prevent distal embolization, though further dilatation may cause symptoms due to nerve compression as well as cosmetic problems. In the present case, an important consideration was graft routing over the elbow joint, which was required because of the large brachial aneurysm found at the anterior aspect of that joint. In such cases, the course of the graft should be carefully determined to prevent its occlusion due to elbow flexion. We think tunneling along the ulnar side of the elbow is an optimal strategy, as that can minimize the influence of elbow flexion on the graft. Moreover, instruction can be given to the patient to prohibit excess and frequent elbow flexion so as to decrease the risk of graft occlusion for a long duration.

## CONCLUSION

We report a surgical case of a true brachial aneurysm associated with AVF for hemodialysis. When a large brachial aneurysm is found at the anterior aspect of the elbow joint, resection followed by revascularization using a reversed great saphenous vein graft routed through the ulnar side of the elbow is considered to be an optimal strategy.

## CONFLICT OF INTEREST STATEMENT

None declared.

## FUNDING

None.

## ETHICAL APPROVAL

The patient consented to the publication of this report.

## DATA AVAILABILITY

Data for this study are available from the corresponding author upon reasonable request.
